# The Power of Movement: A Comprehensive Case Study of Physiotherapeutic Approaches in Electrical Injury Rehabilitation

**DOI:** 10.7759/cureus.64615

**Published:** 2024-07-15

**Authors:** Medhavi V Joshi, Pallavi Bhakne, Chaitanya A Kulkarni, Tushar J Palekar, Pratik Phansopkar

**Affiliations:** 1 Musculoskeletal Physiotherapy, Dr. D. Y. Patil College of Physiotherapy, Dr. D. Y. Patil Vidyapeeth, Pune, IND; 2 Cardiorespiratory Physiotherapy, Dr. D. Y. Patil College of Physiotherapy, Dr. D. Y. Patil Vidyapeeth, Pune, IND; 3 Community-Based Rehabilitation, Dr. D. Y. Patil College of Physiotherapy, Dr. D. Y. Patil Vidyapeeth, Pune, IND; 4 Public Health, Datta Meghe Institute of Higher Education & Research, Wardha, IND; 5 Physical Medicine and Rehabilitation, Dr. D. Y. Patil College of Physiotherapy, Dr. D. Y. Patil Vidyapeeth, Pune, IND; 6 Musculoskeletal Physiotherapy, Ravi Nair Physiotherapy College, Datta Meghe Institute of Higher Education & Research, Wardha, IND

**Keywords:** rehabilitation, neurological impairment, shock, fracture dislocation, shoulder

## Abstract

Electrical injuries are common phenomena in developing countries, due to inadequate safety measures followed during day-to-day electrical repairs. Workplace injuries account for 20% of these. In some severe cases, electrical injuries lead to burns, indirect fracture dislocations, speech impairments, etc. Falls due to electrical injuries leading to secondary complications are very common and, even though not very severe, they do require immediate treatment and adequate rehabilitation.

A 53-year-old male suffered a shoulder injury following an electrical shock. The patient also experienced irritation and speech disturbances. Examination revealed a reduced range of shoulder joints and tightness of muscles of the shoulder complex. Physiotherapy intervention included counseling for the patient and his family members, energy conservation methods for ease in daily activities, a rehabilitation protocol, and modified music therapy. Outcome measures used to assess the progression constituted the Shoulder Pain and Disability Index (SPADI), the Tampa Scale for Kinesiophobia (TSK), and the Depression and Anxiety and Stress Scale. Rehabilitation with adjunct therapy is effective in the overall improvement of the patient’s condition concerning their mental health as well as physical health by early strength training.

## Introduction

Electricity has been a fundamental part of modern life, and this luxury comes with the cost of facing severe life-altering injuries when standardized guidelines for safety are not followed. Electric shock, often a neglected danger, can lead to cardiac complications due to burns, secondary physical injuries, and neurological impact on both central and peripheral nervous systems [1]. Loss of consciousness and peripheral neuropathy have a prevalence of 21-67% and 17% respectively. Apart from the employees working in major industries exposed to electricity while performing their daily activities, daily wage workers like electricians are also highly susceptible to electrocution [2].

Deaths due to falls from height and cardiac rhythm alterations leading to cardiac arrest are the most alarming complications post-electric shock [3]. The extent of injury in an individual affected by shock depends on the duration of the flow of current, the magnitude of the current, and the site of entry. Electric shock that passes through the transthoracic region interferes with the cardiac sinus rhythm leading to fatal respiratory arrest or cardiac damage. Other serious injuries include seizures, paralysis, and brain injuries. Voltage injuries also lead to changes in protein configuration that debilitate the cell wall integrity and functions, as described in various microscopic studies [4].

Physical injuries resulting from falls, immediately post-electrocution, often due to unfavorable individual position, also result in contusions, fractures, and mild soft tissue injuries. Fall from a height on a firm surface post-electric shock in electricians accounted for 35% of cases in a study from India. Studies have also reported rare cases of isolated scapular fractures or bilateral involvement of all the shoulder joint complexes. Studies state that strong tetanic muscular contractions are a facilitating factor for fracture. Fall from height adds to the impact of tetanic muscular contraction involving multiple joints. Pain post-trauma is a function of both direct injury from electric shock and indirect injury from fracture-dislocations and falls. Pain management thus becomes an important aspect while treating the affected individuals [5].

Before the initiation of physical therapy rehabilitation, pain management is done using opioids, acetaminophen, anti-depressants, and nonsteroidal anti-inflammatory drugs. The duration of rehabilitation depends on the severity of the injuries. Hot fomentation, massage therapy, and transcutaneous electrical nerve stimulation are common non-pharmacological modalities used in rehabilitation. Simultaneous management of secondary injury is done using the best evidence-based practice available for the condition.

## Case presentation

The patient was a 53-year-old male who attempted to replace an electric bulb at home three months ago. Without anticipating the perilous danger of shock, he stepped on a chair and, as he approached the bulb with both his upper extremities, he experienced sudden pain and intense paresthesia shot throughout his body. The impact felt by the patient was very strong and he was thrown away by its force, resulting in a fall from the chair. He became unconscious immediately after the fall and was admitted within two hours following the incident. He regained consciousness with severe pain in his left shoulder and a lack of comprehension. He also experienced slurred speech. His previous medical records during hospitalization revealed generalized weakness all over the body for the first few weeks. Nutritional deficiency and shock were found to be the major causes.

The patient underwent a series of laboratory and radiological investigations. While laboratory investigations showed no obvious abnormality, a left shoulder X-ray revealed a posterior dislocation. A neurological consultation was also taken for the patient due to a history of unconsciousness immediately after the fall, and shoulder spica was applied. Medications like tablets Etojet 90 mg, Rabwin D, Calvin, and Vitexid, along with a lignocaine gel, were also prescribed. A nerve conduction study was done with no abnormality seen. The patient was then discharged from the emergency department. The cast was removed after six weeks. Following the removal of the cast, the patient was referred to the physiotherapy outpatient department.

Clinical findings

On the day of consultation with the physiotherapy department, the patient was fully conscious and oriented to time, place, and person. The vitals of the patient were measured to ensure hemodynamic stability. The patient presented with complaints of pain in the neck, and posterior aspect of the left shoulder, which was radiating down the left arm, and restrictions in performing activities of daily living, such as grooming, eating, and in-bed transfers. In addition to these complaints, the patient expressed his increased frustration, irritability, occasional double vision, short-term memory loss as well as slurred speech, which had been present from the day of injury. On observation, the patient was irritated, and unwilling to move the hand. He was supporting the forearm of the left side with his right hand. The left shoulder was slightly elevated with reduced lordotic curvature of the cervical spine. On palpation, there was a positive jump sign indicating grade-three tenderness. On further examination, tightness of the suboccipital, trapezius, pectoralis and levator scapulae, and scaleni were present. Post-fracture-dislocation assessment for the shoulder joint complex was done, revealing painful reduced active and passive ranges on the affected side (Table 1).

**Table 1:** Range of motion of the shoulder pre- and post-treatment

Joint/range	Active range (week 1) (in degrees)	Active range (week 4)
Shoulder flexion	0-35	0-110
Shoulder extension	0-5	0-15
Shoulder abduction	0-47	0-100
Shoulder adduction	47-0	100-0

All ranges for the unaffected shoulder complex and cervical spine were complete within normal functional ranges. Scapular movement assessment revealed alterations in the scapulothoracic rhythm. Lateral rotation of the scapula guided by serratus anterior was reduced. The normal position of the scapula on the thorax was altered. On muscle strength examination, the middle trapezius and rhomboids were found to be weak. Cervical flexors, extensors, and rotators were graded fair on the muscle strength examination. Special tests for rotator cuff injury were not positive.

Investigations 

**Figure 1:**
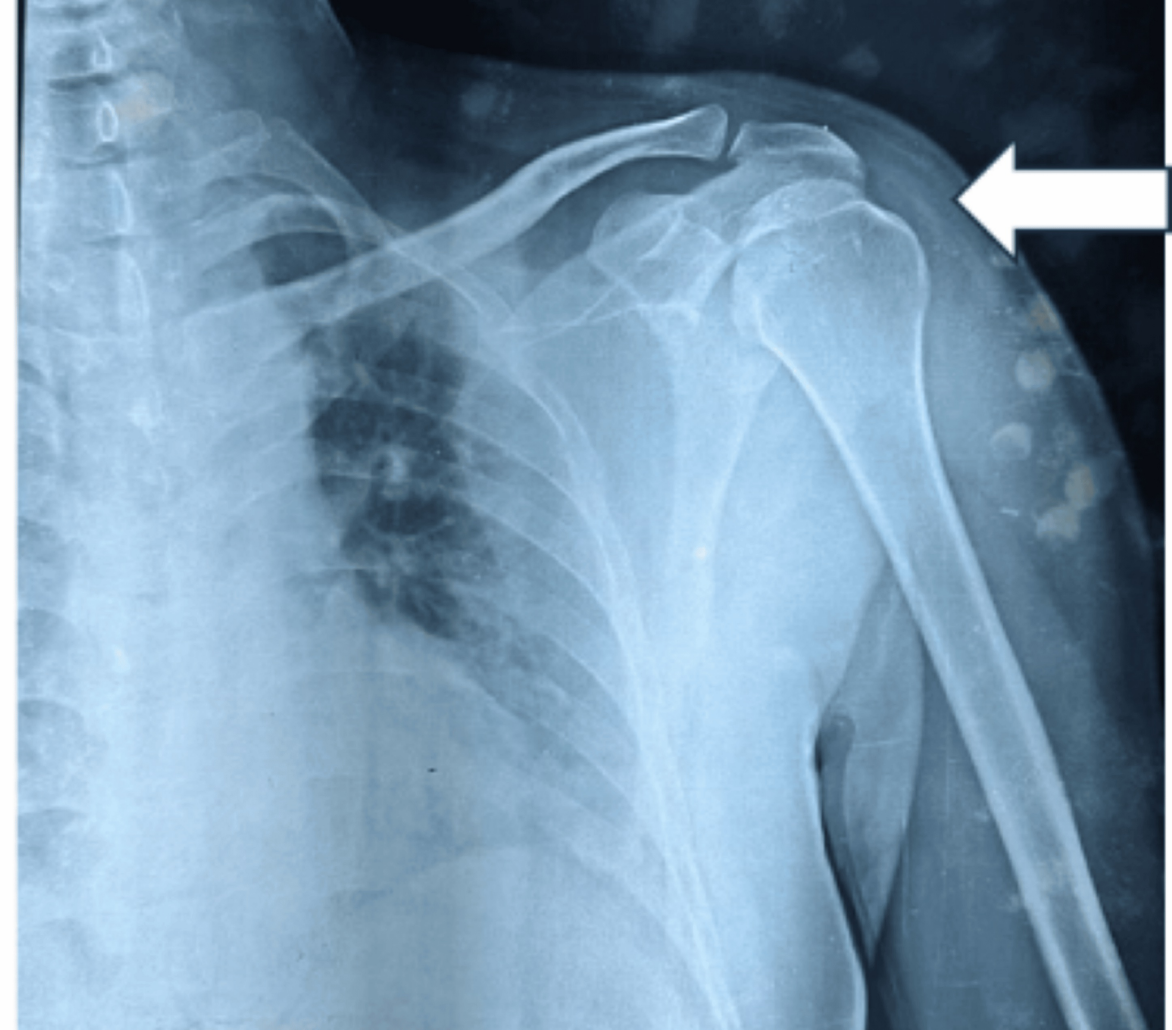
Left shoulder X-ray suggestive of posterior dislocation (white arrow)

A previous shoulder X-ray revealed posterior dislocation of the left shoulder (Figure 1). MRI of the left shoulder joint was done after cast removal, which revealed mild capsular and synovial thickening around the acromioclavicular joint, focal depression in the anteromedial portion of the left humeral head, a fairly well-defined, lobulated, intra-medullary lesion in the head for humerus suggestive of enchondroma, mild changes of adhesive capsulitis, degenerative signals in the superior glenoid labrum, fluid collection along the tendon of the long head of biceps in bicipital groove, and sub-acromial and sub-deltoid bursitis.

Treatment

After the removal of the cast, rehabilitation protocol was started in the outpatient department setting. The patient was consulted during the first session for kinesiophobia as it could lead to complications of immobilization and prolong the period of recovery. Also, to reduce the burden of neuropsychological sequelae, the patient and the caregiver were made aware of the condition and the expected time of recovery. Relaxation exercises were also a part of patients' daily protocol. Box breathing, progressive muscle relaxation, and a fidget spinner were used alternatively for providing relaxation with breath control [6]. Passive stretches for pectoralis major, pectoralis minor, upper trapezius, and scalene muscles were given with a 45-second hold and five repetitions per session for the first 10 days followed by advising the patient to perform active stretches as a home protocol every day for 10 days with the same frequency and hold timing.

Maitland mobilizations to improve all ranges of the glenohumeral joint were given along with scapular mobilization. Scapular mobilization was altered with myofascial release of the subscapularis and other muscles that stabilize the scapula. Isometric exercises to maintain muscle integrity were performed with the patient sitting upright in a chair with hand supported, elbow at 90 degrees and feet touching the ground. The first instruction given prior to the initiation of isometric contraction (shoulder extension, abduction, internal rotation, external rotation, and flexion) was to inhale for three seconds and exhale for six seconds, thrice, to induce muscle relaxation.

The isometric contraction was performed against the pulley resistance of 0.5-kilogram weight after five days of initial management of pain. The pain was reduced by 40% and the willingness to move the hand also improved when resistance training was started. Progression of strength training was done by increasing the weight by 0.5 kilograms once 10 RM (repetition maximum) was achieved. Postural training using biofeedback sensors for relaxing the upper trapezius was given on the first day to familiarize the patient with the feeling of active, relaxed, and overactive muscle. Postural training under supervision was discontinued after five sessions and home protocol including chin tucks, isometrics for cervical muscles, and stretches were recommended to the patient.

Music therapy was used as an intervention to manage anxiety and pain [7]. Since music therapy is used as a part of rehabilitation for speech in children with developmental delay, we, in this case, incorporated it as an adjunct intervention for the treatment of adult speech disability post-electrical shock. This specific means of communication and expression stimulates the ability of an individual to facilitate communication. The improvements we see result from activating listening, perception, and processing of sounds and musical structures [8]. It also improves phonological memory (PGN) and understanding of sentences [9].

Outcome measures

The prognosis of the patient is objectively assessed by using various outcome measures. The Shoulder Pain and Disability Index (SPADI), the Tampa Scale for Kinesiophobia (TSK), and the Depression and Anxiety and Stress Scale were used in the study [10,11]. Outcome measures were taken on day one pre-treatment and post six weeks after treatment (Table 2). A telephonic follow-up was also done to check the long-term effect and progression.

**Table 2:** Progression in the patient's conditions as indicated by outcome measures on day one and after six weeks SPADI: the Shoulder Pain and Disability Index; TSK: the Tampa Scale for Kinesiophobia

Outcome measure	SPADI	TSK	Depression and Anxiety and Stress Scale		
Week 1 (pre-treatment)	42 (pain), 65 (disability)	54	Depression: 25	Anxiety: 18	Stress: 27
Week 6	25 (pain), 42 (disability)	36	Depression: 10	Anxiety: 7	Stress: 11

## Discussion

Electrical injuries occur very commonly and the impact can be minor or fatal depending on the voltage and length of the current. This case study of electrical injury focuses on a holistic approach to treatment. A tailored protocol was found to be the most effective practice. Several research studies published on burn post-electrical injury management include the use of orthosis in case of third-degree injury [12]. Orthosis has also helped in maximizing mobility in such cases. Another study used thermal biofeedback where the temperature of the affected area was increased by certain degrees to reduce pain [13]. Ketenci et al., in their case report of bilateral shoulder dislocation post electric shock, reported the initiation of rehabilitation by performing active and passive range of motion, and capsular stretching [14]. Results of a few studies suggest that early physical examination, radiographic investigations, proper immobilization, and rehabilitation result in complete functional recovery.

Another case report has described that early detection of bilateral shoulder dislocation through investigations helped in identifying a brain tumor through a lesion [14]. Life-threatening incidents or death due to electrocution also occur after a latent period, during which the patient may seem to be recovering [15]. Muscle necrosis following high-pressure electrical shocks as a result of hypoxia is a severe complication. Standardized physiotherapy care in post-burn recovery cases should be considered a basic but pertinent part. In this case, music therapy was a major accelerating factor in recovery. Since enfeebling communication makes the patient more reserved from being involved in any conversations or social activity [16], this further affects their mental well-being. World Health Organization also states that the complex continuum of mental health and disease needs to be addressed irrespective of the physical injury to attain maximum patient recovery and functional outcome. We used music therapy on several levels. A cumulative effect of improved exercise adherence, reduced stress, and mildly improved speech was observed.

## Conclusions

Electrical injuries related to low voltage have a significant effect on patients' musculoskeletal, neurological, and psychological systems, affecting their ability to return to work. Adequate training about the use of preventive measures, such as insulated materials, and protective devices, and following safety measures was given to the patient to reduce the incidence of injuries related to electrocution, especially at the patient's workplace. Early detection of the multifactorial symptoms and adequate management helped in the early recovery of our patient.

As part of physiotherapy rehabilitation, the protocol focused on helping the patient gain functional independence, reducing kinesiophobia, and relaxation to reduce any neurological irritation associated with movement. Breathing control and relaxation instruments were coordinated with strength training and they proved to be beneficial in keeping the patient's compliance with the treatment. Electrocuted patients with neurological symptoms should be managed with appropriate counseling and relaxation techniques that reduce the occurrence of episodes of irritation due to exercise cues.
